# Diversification of signal identity and modus operandi of the *Haemophilus influenzae* PAS-less ArcB sensor kinase

**DOI:** 10.1371/journal.pone.0315238

**Published:** 2024-12-05

**Authors:** Adrián F. Alvarez, Antonio de Jesús Santillán-Jiménez, Eder Flores-Tamayo, Juan L. Teran-Melo, Oscar J. Vázquez-Ciros, Dimitris Georgellis

**Affiliations:** 1 Departamento de Genética Molecular, Instituto de Fisiología Celular, Universidad Nacional Autónoma de México, México City, México; 2 Department of Microbiology, Immunology and Molecular Genetics, University of Kentucky, Lexington, KY, United States of America; Gandhi Insititute of Technology and Management, INDIA

## Abstract

Bacteria employ two-component signal transduction systems (TCS) to sense environmental fluctuations and adjust their cellular functions. The Arc TCS is crucial for facultative anaerobes as it enables adaptation to varying respiratory conditions. The *Escherichia coli* ArcB detects redox changes through two cysteine amino acid residues within its PAS domain. However, the ArcB homologs from most bacteria belonging to the Pasteurellaceae family, lack the entire PAS domain, and in consequence the two regulatory cysteine amino acid residues. In this study, we show that the PAS-less ArcB of *Haemophilus influenzae* regulates its activity via a cysteine-independent mechanism, and we provide data suggesting that it responds to metabolic signals rather than redox cues. Thus, these two ArcB orthologs sense distinct signals and their regulatory mechanism rely on different molecular events. Our findings reveal divergent evolutionary trajectories of these ArcB homologs, despite the overall conservation of protein components, providing an example of how evolution has shaped different sensing strategies in bacteria.

## Introduction

Cells are equipped with sophisticated mechanisms to detect and respond to diverse environmental changes. In bacteria, these mechanisms depend primarily on the two-component signal transduction systems, which consist of sensor histidine kinase (HK) and response regulator (RR) proteins that communicate through phosphorylation and dephosphorylation events, utilizing histidine and aspartate residues as phosphoryl-group donors and acceptors [1]. Although these systems are present in certain eukaryotes, including plants and fungi, they are significantly more prevalent in prokaryotes [2]. Signal transduction by these systems leads to changes in gene expression, modulating metabolic processes and cellular behavior, and allowing organisms to adapt to fluctuating environments [3].

**Fig 1:**
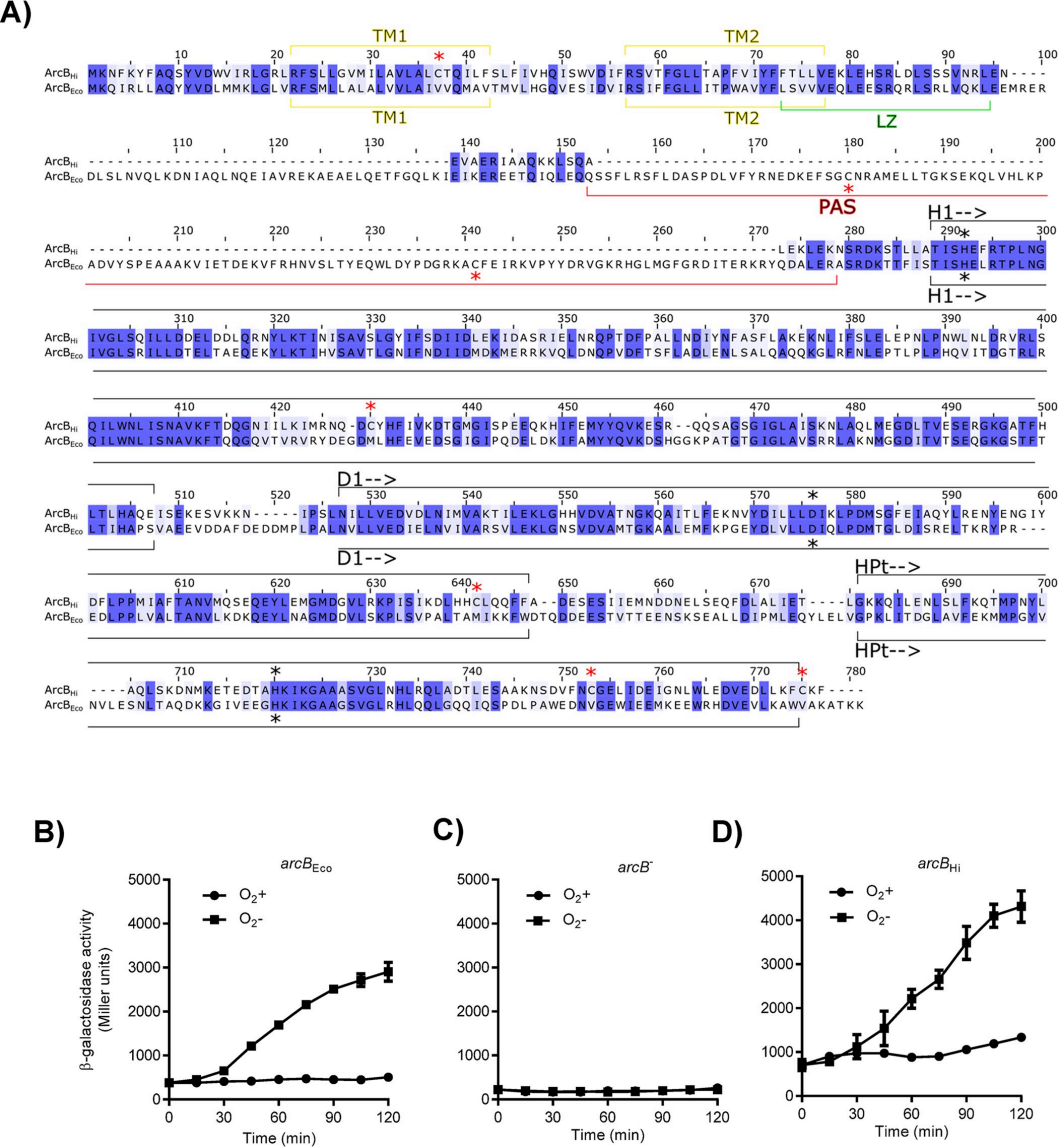
PAS-less ArcB_Hi_ expression restores ArcA-P-dependent regulation of *cyd-lacZ* reporter expression in a Δ*arcB E*. *coli* mutant strain. A) Sequence alignment of ArcB_Hi_ with ArcB_Eco_. Identical amino acids are shaded in dark blue, and related residues in light blue. Protein domains or modules in each ArcB sequence are delimited with brackets, and labeled as follow; TM, transmembrane domain; LZ, leucine zipper; PAS, PAS domain; H1, transmitter domain; D1, receiver domain; HPt, phosphotransfer domain. The position of phosphorylatable His and Asp residues are indicated by black asterisk, whereas the position of relevant Cys residues in ArcB homologues are indicated by red asterisks. B-D) Cultures of strain ECL5003 (*arcB*_Eco_) (B) and its isogenic strains ECL5004 (Δ*arcB*) (C) and ECL5004 harboring plasmid pEXT22arcBHi (*arcB*_Hi_) (D), all carrying the ArcA-P–activatable λФ(*cydA*’*-lacZ*) reporter, were grown aerobically in buffered LB medium (pH 7.4) supplemented with 20 mM D-xylose. At an OD600 of 0.2, an aliquot was taken to measure the β-galactosidase activity (designated as 0 min), and the remaining culture was split into two parts. One part was maintained under aerobic conditions (circles) as a control, while the other was shifted to anaerobiosis (squares), and the β-galactosidase activity was monitored over time. The data represent the averages from three independent experiments, with standard deviation values indicated by error bars.

The Arc (anoxic redox control) TCS is a well-conserved signaling system in γ-Proteobacteria. It plays a crucial role in the intricate transcriptional regulatory network that allows facultative anaerobic bacteria to detect and adapt to varying respiratory growth environments [4–8]. The Arc system of *Escherichia coli* (Arc_Eco_) consists of the membrane-anchored ArcB_Eco_ HK and the cytosolic ArcA_Eco_ RR [9, 10]. ArcA_Eco_ is a typical response regulator with an N-terminal receiver domain containing a conserved Asp residue at position 54 and a C-terminal helix-turn-helix DNA-binding domain. In contrast, the ArcB_Eco_ protein is a hybrid HK, containing two transmembrane segments that delimit a short periplasmic domain, which is not directly involved in signal perception [11], and three cytosolic catalytic domains: a transmitter domain (H1), a receiver domain (D1) and a histidine phosphotransfer domain (HPt), with a conserved His292, Asp576, and His717 respectively [10, 12]. Additionally, the ArcB_Eco_ protein contains a functional leucine zipper [13] and a Per-Arnt-Sim (PAS) domain [14], which includes two redox-active cysteines [15]. These elements are located in the linker region that connects the second trans-membrane domain with the transmitter domain. Under reducing growth conditions, ArcB_Eco_ autophosphorylates at the expense of ATP, a process enhanced by certain anaerobic metabolites such as D-lactate, acetate, and pyruvate [16, 17], and transphosphorylates ArcA_Eco_ via a His292→Asp576→His717→Asp54 phosphorelay [18, 19]. Phosphorylated ArcA (ArcA-P) represses the transcription of several operons associated with respiratory metabolism, while activates those that encode proteins for fermentative metabolism [8, 20–22]. Under aerobic growth conditions, ArcB_Eco_ functions as a specific ArcA_Eco_-P phosphatase, catalyzing the dephosphorylation of ArcA_Eco_-P through a reverse Asp54→His717→Asp576→Pi phosphorelay [23, 24], resulting in the silencing of the system. The molecular mechanism of ArcB_Eco_ regulation involves the oxidation/reduction of two PAS-located cysteine residues, which form intermolecular disulfide bonds [15], using the quinone electron carriers as direct oxidants or reductants [25–28]. Curiously, ArcB homolog proteins from most bacteria in the family Pasteurellaceae, such as *Haemophilus*, *Mannheimia*, *Pasteurella*, and *Actinobacillus* species, lack the entire PAS domain, including the two regulatory cysteine residues (Fig 1A) [29–32]. Despite this, the ArcB of *Haemophilus influenzae* (ArcB_Hi_) was shown to functionally complement a Δ*arcB E*. *coli* strain, and, also, to *in vitro* transphosphorylate ArcA_Eco_ (Manukhov et al. 2000, Georgellis et al. 2001b), suggesting a high level of functional and structural similarity between these Arc ortholog proteins.

Here, we used ArcB_Hi_ to address the question of whether the PAS-lacking ArcB homologs do respond to redox changes in a similar manner to their *E*. *coli* counterpart. Our results reveal that this is not the case, and that distinct signaling molecules and regulatory mechanisms govern the activity of the two γ-proteobacterial ArcB species, highlighting an evolutionary divergence in this signal transduction pathway, despite the overall conservation of the protein components. Such divergence enables systems with a common evolutionary origin to sense and respond to distinct environmental stimuli, that is adapting them to the specific ecological niche of each bacterial species.

## Materials and methods

### Bacterial strains and culture conditions

The *E*. *coli* strains used in this study have the genetic background of the *E*. *coli* MC4100 reference strain [33]. *E*. *coli* cells were routinely cultured in LB medium at 37°C. When necessary, kanamycin, tetracycline or chloramphenicol was used at final concentrations of 50, 12.5 or 34 μg ml-1, respectively. For β-galactosidase activity assays, the λФ(*cydA*’*-lacZ*) bearing strains ECL5003 [MC4100 Δ*fnr*::Tn*9*(Cm^r^) λФ(*cydA*’*-lacZ*)] and ECL5004 [MC4100 Δ*arcB*::Tet^r^ Δ*fnr*::Tn*9*(Cm^r^) λФ(*cydA*’-*lacZ*)] [11] were grown in LB containing 0.1 M MOPS (pH 7.4). When needed, media were supplemented with 20 mM D-xylose, D-lactate, L-lactate, pyruvate, acetate or formate. When indicated, dithiothreitol (DTT) was added to the cultures to a final concentration of 10 mM. To assess *in vivo* phosphatase activity of ArcB, HK-independent ArcA-P was generated by growing cells in a defined minimal medium [1 mM KH2PO4, 40 mM KCl, 34 mM NaCl, 20 mM (NH4)2SO4, 1 μM FeSO4, 0.3 mM MgSO4, 1 μM ZnCl2, 10 μM CaCl2, and 0.1 M MOPS, at a final pH of 7.4] supplemented with 20 mM pyruvate as described previously [24].

### Plasmids and oligonucleotides

To construct the low-copy number plasmid pEXT22arcBHi, a DNA fragment containing the *arcB*_*Hi*_ coding sequence with its native promoter, was amplified by PCR using primers HIAB-N (5’- ACTGAATTCTGGATATGGTAAATCGGG-3’) and HIAB-3’ (5’- CCCGGATCCATGCACCCATTTTAAGCCTC-3’), and the chromosomal DNA of *H*. *influenzae* Rd strain KW20 as a template. The PCR product was digested with EcoRI and BamHI and cloned into EcoRI-BamHI-digested pEXT22 [34], resulting in the plasmid pEXT22arcBHi. ArcBHi punctual mutants were created by site-directed mutagenesis according to a two-step PCR procedure [35]. The first PCR amplifications were performed using the plasmid pEXT22arcBHi as a template and the primers pair HIB-C472A-Fw (5’-GGATTTACACCATGCTCTACAGCAATTTTTTGCG-3’) / HIAB-3’ or HIAB-N / HIB-C37A-Rv (5’-GACTAAATAAAATCTGAGTAGCAAGAGCTAAAACCGCGAG-3’), for C472A or C73A replacement, respectively. Each purified product was used as a primer with primer HIAB-N or HIAB-3’ for the second PCR, using the plasmid pEXT22arcBHi as a template. The second PCR products were digested with EcoRI and BamHI and cloned between the corresponding sites of vector pEXT22, resulting in pEXT22arcBHi-C472A and pEXT22arcBHi-C37A.

### β-galactosidase activity assay

For aerobic growth, *E*. *coli* cells were cultured in 10–50 ml of medium in 250-ml baffled flasks at 37°C with shaking (300 rpm). The aerobic / anaerobic shift experiments were carried out as previously described [26]. Briefly, for the shift from aerobiosis to anaerobiosis, cells grown aerobically to an OD600 of 0.2 were transferred to pre-warmed screw-capped tubes filled to the brim and stirred with a magnet. The remaining aerobic culture was further incubated with shaking at the same temperature. Samples were taken from the anaerobic screw-capped tubes and aerobic baffled flasks at specified times. For the anaerobiosis to aerobiosis shift experiment, cells in screw-capped tubes at 37°C were grown to an OD600 of 0.2, transferred to pre-warmed baffled flasks, and incubated with shaking. Samples were taken at specified times from both aerobic and anaerobic cultures. β-galactosidase activity was measured and expressed in Miller units [36].

## Results

### Regulation of *H*. *influenzae* ArcB occurs via a cysteine-independent mechanism

**Fig 2:**
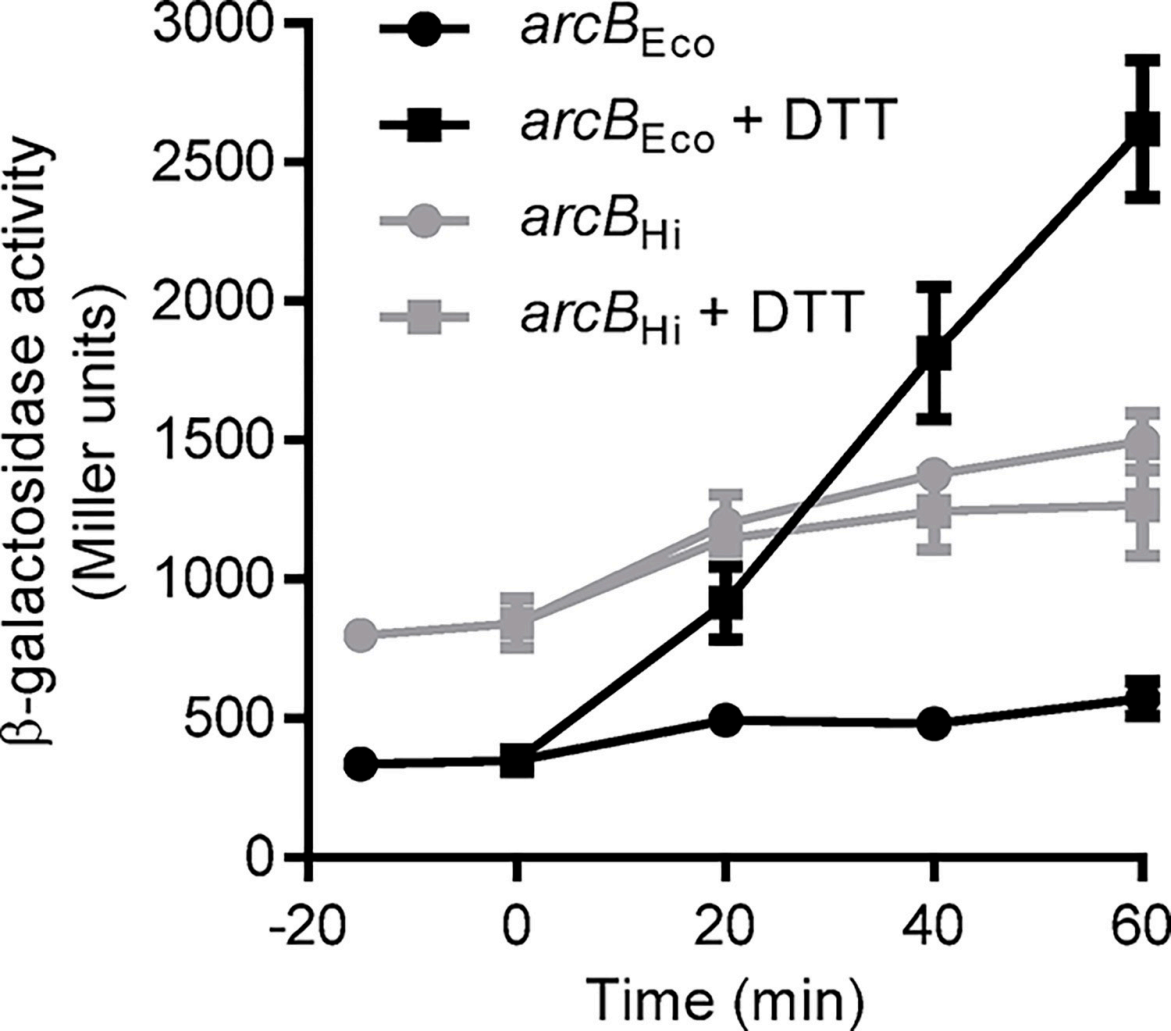
Effect of the reductant agent DTT on the ArcB_Eco_ and ArcB_Hi_ aerobic activity. Cells of strain ECL5003 (*arcB*_Eco_) (black) and of it isogenic ECL5004 harboring plasmid pEXT22arcBHi (*arcB*_Hi_) (gray) were grown aerobically in LB medium supplemented with 0.1 M MOPS (pH 7.4) and 20 mM D-xylose. When the culture reached an OD600 of 0.2, a sample was taken to measure β-galactosidase activity (depicted as -20 min). At time 0 min, the culture was split into two parts: one continued as an aerobic control (circles), while DTT was added to the other to a final concentration of 10 mM (squares), and β-galactosidase activity was monitored over time. The data represent the averages from three independent experiments, and the standard deviation values (error bars) are indicated.

**Fig 3:**
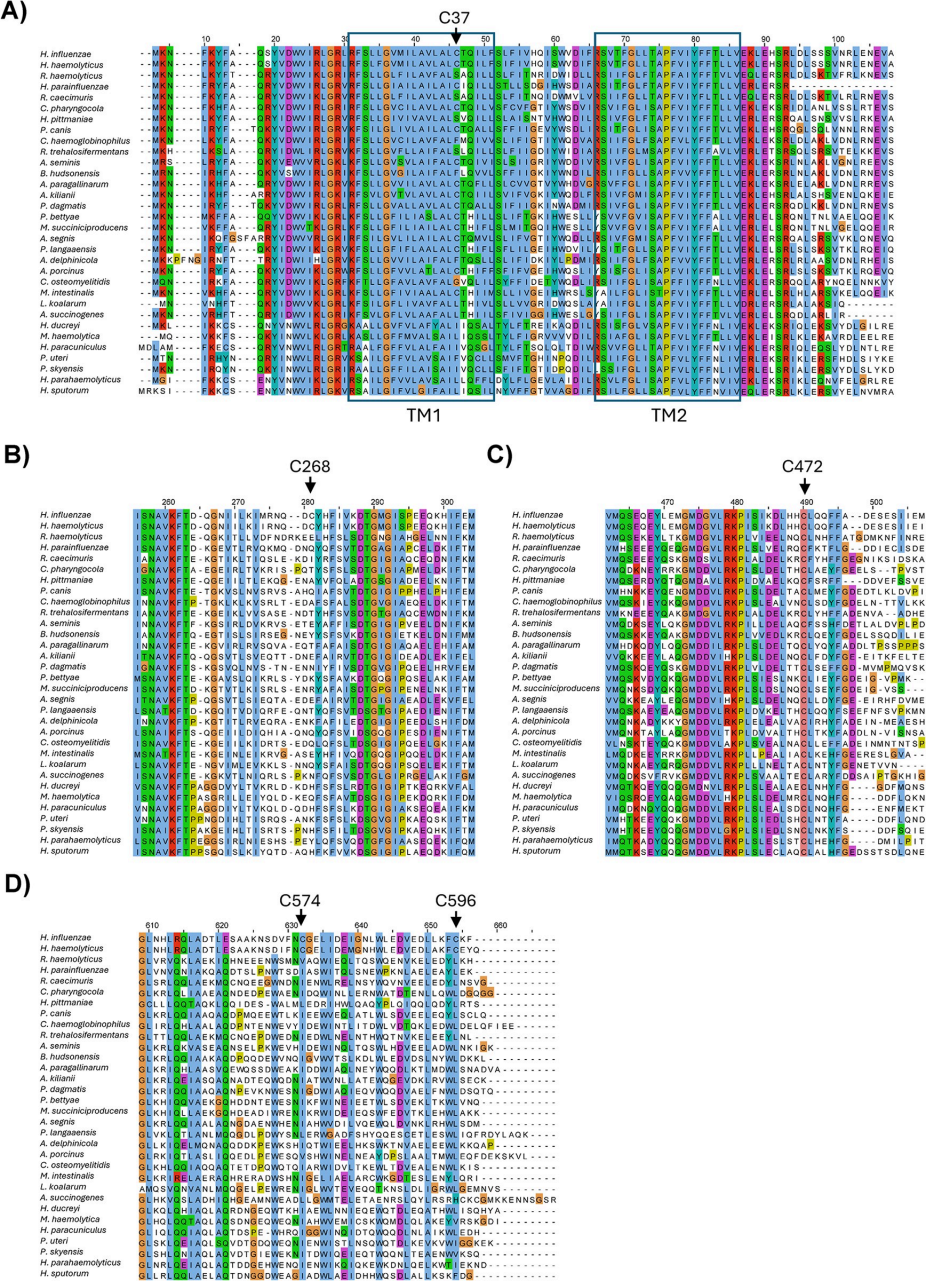
Conservation of the ArcB_Hi_ cysteine residues across the PAS-less ArcB orthologs. Multiple sequence alignment of the 32 nonredundant PAS-less orthologs (S1 Table). For clarity, only four sections of the alignment are shown, corresponding to Cys37 (A), Cys268 (B), Cys472 (C), and Cys574/Cys596 (D) of ArcB_Hi_.

**Fig 4:**
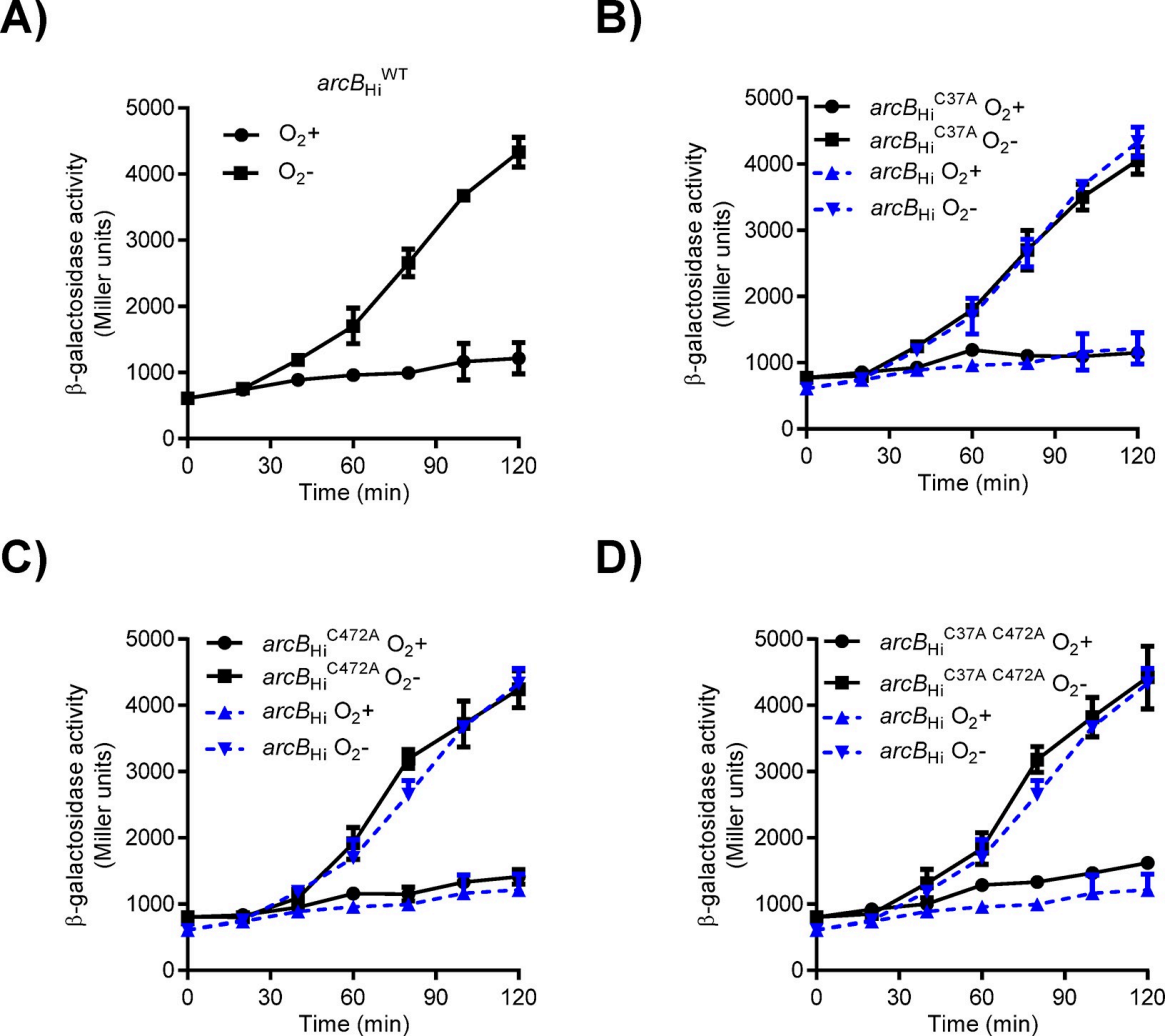
Cysteine-independent regulation of the ArcB_Hi_ activity. Cultures of strain ECL5004 (Δ*arcB*) harboring either plasmid pEXT22arcBHi (*arcB*_Hi_) (A), pEXT22arcBHi-C37A (*arcB*_HiC37A_) (B), pEXT22arcBHi-C472A (*arcB*_HiC472A_) (C), or pEXT22arcBHi-C37A-C472A (*arcB*_HiC37A C472A_) (D), all carrying the λФ(*cydA*’*-lacZ*) reporter, were grown aerobically in LB medium buffered with 0.1 M MOPS (pH 7.4) and supplemented with 20 mM D-xylose. Following the shift to anaerobic conditions, samples were collected, and β-galactosidase activity was measured as in Fig 1B–1D. To facilitate comparisons, the ß-galactosidase activities of the strain expressing wild type *arcB*_Hi_, shown in panel A, is also included in panels B, C and D (blue lines).

Previous reports demonstrated that heterologous expression of the *arcB* gene from *H*. *influenzae* (*arcB*_Hi_) restores the anaerobic repression of the ArcA-P repressible reporters *lldP-lacZ* and *sdh-lacZ* in Δ*arcB E*. *coli* mutant strains [29, 30]. To investigate whether this PAS-less ArcB_Hi_ protein is able to rapidly respond to redox changes, we monitored the Arc activity in cells shifted from non-stimulatory to stimulatory growth conditions. To this end, the *cydA-lacZ* operon fusion carrying *E*. *coli* strains ECL5003 (Δ*fnr*), ECL5004 (Δ*arcB* Δ*fnr*) and ECL5004 harboring pEXT22arcBHi, a low-copy-number plasmid expressing ArcB_Hi_, were grown aerobically in buffered LB supplemented with D-xylose. The *fnr* mutation was employed to avoid Fnr-dependent repression of the reporter, and D-xylose was used as a supplement because it promotes anaerobic growth while minimizing catabolic repression [37]. At an OD600 of approximately 0.2, the cell cultures were divided into two. One part was maintained under aerobic conditions, while the other was shifted to anaerobic conditions, and the β-galactosidase activity was followed over time. As expected, moving the aerobic culture to anaerobiosis led to a prompt increase in reporter expression in the *E*. *coli* wild-type strain (Fig 1B), indicating proper activation of ArcB_Eco_ kinase activity. In contrast, the *arcB* mutant strain showed no increase in reporter expression (Fig 1C). Accordingly, when the *arcB* mutant was complemented with a low-copy-number plasmid carrying the *arcB*_*Hi*_ gene, reporter expression increased progressively after the shift to stimulatory conditions, reaching 1.3-fold higher value than the one of the wild type strain (ArcB_Eco_) (Fig 1D). It, thus, appears that ArcB_Hi_ responds promptly to the anoxic growth conditions. Curiously, under non-stimulatory conditions, the basal expression level of the *cyd-lacZ* reporter in cells expressing ArcB_Hi_ was approximately two-fold higher compared to cells harboring ArcB_Eco_ (Fig 1D). This suggests that ArcB_Hi_ might retain some residual activity even under aerobic conditions. In ArcB_Eco_, modulation of the kinase and phosphatase activities depends on the redox state of two Cys residues, located within its PAS domain, which is absent in the ArcB_Hi_ ortholog. On the other hand, the primary sequence of ArcB_Hi_ includes five cysteine residues that could potentially be involved in the regulation of its kinase activity (Fig 1A). To explore this possibility, we compared the effect of the membrane-permeable reducing agent dithiothreitol (DTT) on the aerobic activity of ArcB_Eco_ and ArcB_Hi_. This experiment is based on the fact that the intermolecular disulfide bonds formed by the two Cys residues in ArcB_Eco_ are reduced *in vivo* by thiol-reducing agents that are able to permeate the plasma membrane, such as DTT and 2-mercaptoethanol, resulting in the activation of ArcB as a kinase [15]. As expected, the addition of DTT to an aerobic culture of the wild-type strain resulted to the immediate activation of *cydA-lacZ* reporter expression (Fig 2). In contrast, addition of DTT to *E*. *coli* cells expressing ArcB_Hi_ failed to activate *cydA-lacZ* expression (Fig 2), suggesting that oxidation/reduction of one or more Cys residues in ArcB_Hi_ is unlikely to be the molecular event that regulates its activity. Subsequently, to provide more definite evidence for the above suggestion, we examined the conservation pattern of the Cys residues across the PAS-less ArcB homologs. We acquired 32 non-redundant PAS-lacking ArcB-protein sequences from the GenBank database (S1 Table) and performed a multiple sequence alignment using ClustalW (Fig 3A–3D). The analysis revealed that only Cys472 of ArcBHi was highly conserved among PAS-less ArcB homologs (32/32), while Cys37 was present in 15 out of the 32 ArcB orthologs. In contrast, Cys268, Cys574, and Cys596 of ArcBHi were not conserved in the compared sequences. To examine whether these conserved cysteine amino acid residues are involved in the regulation of ArcB_Hi_, we replaced Cys37 and/or Cys472 with Ala, using site-directed mutagenesis on plasmid pEXT22arcBHi, and the resulting plasmids were introduced into ECL5004 (Δ*arcB cydA-lacZ*). The *E*. *coli* strains harboring the mutant *arcB*_*Hi*_ alleles were grown aerobically and at an OD_600_ of ~0.2 the cultures were divided to two. One half was maintained under aerobic growth conditions whereas the other was shifted to anaerobiosis, and their β-galactosidase activity was followed. It was found that shifting the aerobically grown *E*. *coli* cultures to anaerobic conditions resulted in a rapid increase in reporter expression for all strains containing either wild type or mutated *arcB*_*Hi*_ alleles (Fig 4A–4D). This result supports the conclusion that ArcB_Hi_ employs a regulatory mechanism independent of intermolecular disulfide bond formation/reduction, and it has evolved to respond, most probably, to different signaling molecules.

### Activation of ArcB_Hi_ under aerobic conditions

**Fig 5:**
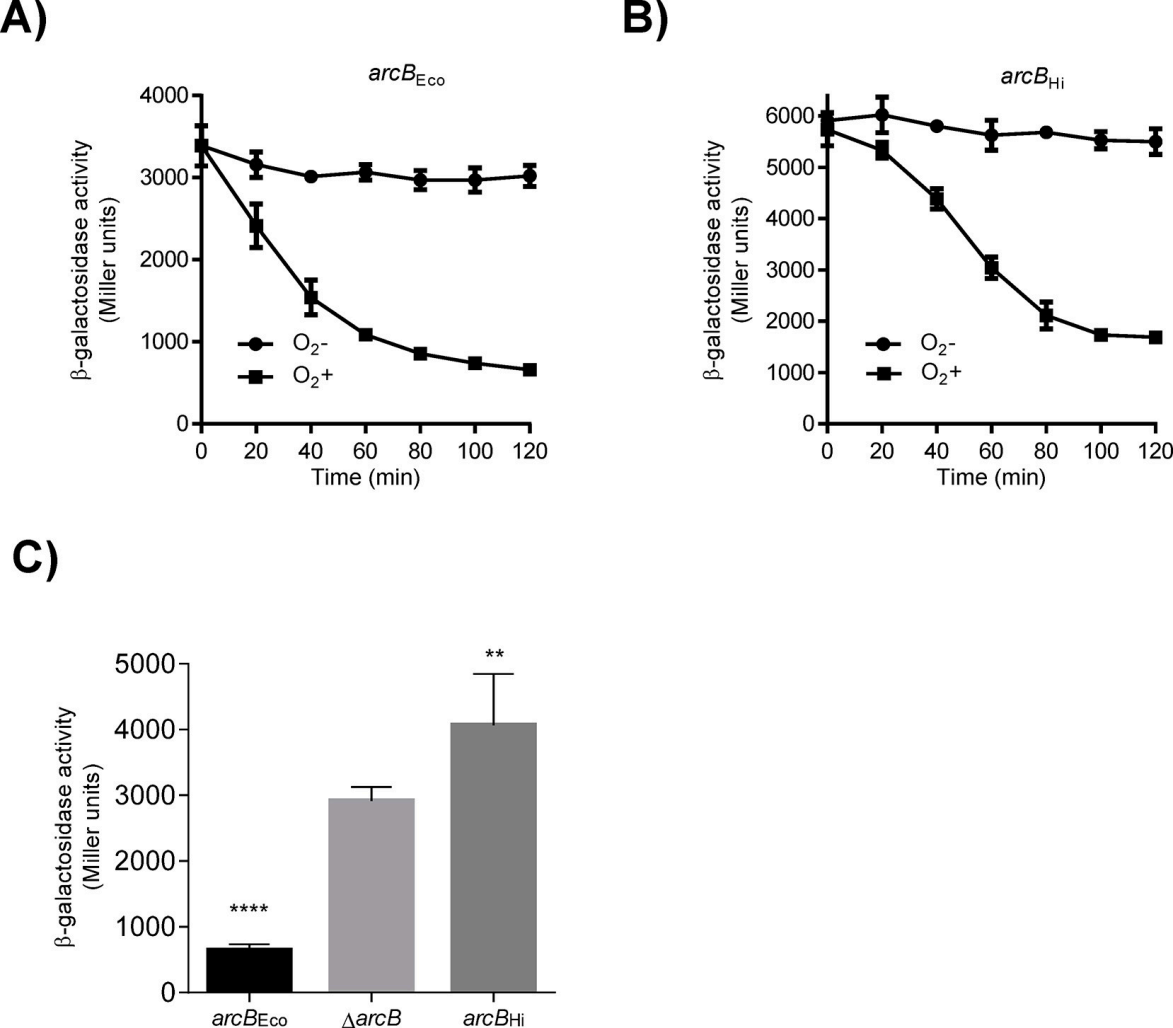
ArcA_Eco_-P phosphatase activity of ArcB_Hi_ under aerobic conditions. A-B) Cells of strain ECL5003 (*arcB*_Eco_) (A) and its isogenic strain ECL5004 harboring plasmid pEXT22arcBHi (*arcB*_Hi_) (B), both carrying the ArcA-P–activatable λФ(*cydA*’*-lacZ*) reporter, were cultured anaerobically in LB medium with 0.1M MOPS buffer (pH 7.4) and 20 mM D-xylose. When the OD600 reached 0.2, an aliquot was taken to measure β-galactosidase activity (marked as 0 min), and the remaining culture was split into two portions. One portion remained under anaerobic conditions (circles), while the other was shifted to aerobic conditions (squares), and the β-galactosidase activity was tracked over time. C) Testing the specific ArcA_Eco_-P phosphatase activity of ArcB_Hi._*in vivo*. Strain ECL5003 (*arcB*_Eco_) (black bars) and its isogenic strains ECL5004 (Δ*arcB*) (light gray bars) and ECL5004 harboring plasmid pEXT22arcBHi (*arcB*_Hi_) (dark gray bars) were grown aerobically with pyruvate as the sole carbon source. At an OD600 of 0.6, β-Galactosidase activity was measured and expressed in Miller units. Data represent averages from three independent experiments, with standard deviations shown, and were analyzed using one-way ANOVA with Holm-Sidak multiple comparisons test (*P* values: **, < 0.01; ****, < 0.0001).

The above results strongly suggest that the regulation of ArcB_Hi_ activity relies on a mechanism distinct from the oxidation/reduction of its Cys residues. Despite this difference, ArcB_Hi_ expression in a Δ*arcB E*. *coli* strain functionally restores the Arc signaling pathway and retains the aerobic/anaerobic regulatory pattern. Nonetheless, we observed that the aerobic activity of ArcB_Hi_ is significantly higher than that of ArcB_Eco_ (Fig 1B and 1C), encouraging us to speculate that the PAS-less ArcB_Hi_ may exhibit delayed and/or incomplete inactivation when the cells are shifted from stimulatory to non-stimulatory conditions. To explore this possibility, we compared the time lag of ArcA-P dephosphorylation following a shift from anaerobic to aerobic growth conditions in *E*. *coli* cells expressing either ArcB_Eco_ or ArcB_Hi_. To this end, strains ECL5003 (Δ*fnr*) and ECL5004 (Δ*arcB* Δ*fnr*) harboring plasmid pEXT22arcBHi (*arcB*_Hi_), both bearing the *cydA-lacZ* reporter, were grown anaerobically in LB medium supplemented with D-xylose. At an OD600 of 0.2, a sample was withdrawn, and the expression of the reporter was measured (Fig 5). As expected, reporter expression was high in both strains, indicative of full ArcB kinase activity. Subsequently, the cultures were either maintained in anaerobiosis or shifted to aerobic conditions, and the time course of the β-galactosidase activity was monitored. It was observed that shifting the anaerobic culture to aerobic conditions caused an almost immediate decrease in reporter expression in the strain carrying ArcB_Eco_, indicating effective ArcA-P dephosphorylation and ArcB/A silencing (Fig 5A). In contrast, when the strain expressing ArcB_Hi_ was shifted to aerobic growth conditions, the decrease in reporter expression was somewhat delayed, and the β-galactosidase activity remained in higher levels by the end of the experiment as compared to the strain expressing ArcB_Eco_ (Fig 5B). This suggests that ArcB_Hi_ may exhibit weaker ArcA_Eco_-P-specific phosphatase activity and/or respond to redox-independent signals. To examine the effectiveness of ArcB_Hi_ to dephosphorylate ArcA-P, we generated ArcB-independent ArcA-P *in vivo*. To this end, the ECL5004 (Δ*arcB* Δ*fnr cydA-lacZ*) strain harboring plasmid pEXT22arcBHi, along with its isogenic ECL5003 (Δ*fnr cydA-lacZ*) and ECL5004 strains, were grown aerobically in defined minimal media supplemented with 20 mM pyruvate as the sole carbon and energy source, and at an OD600 of 0.6 the ß-galactosidase activity was measured (Fig 5C). Pyruvate was selected because previous studies have demonstrated that, these growth conditions favor the accumulation of the intracellular concentration of acetyl-phosphate [38], and in the absence of ArcB, ArcA autophosphorylates at the expense of acetyl-phosphate [13, 39]. As expected, the Δ*arcB* strain exhibited high β-galactosidase activity, indicating proper ArcA phosphorylation and activation of reporter expression, whereas the wild-type strain displayed a 5-fold lower reporter activity (Fig 5C), indicating ArcB_Eco_-dependent ArcA-P dephosphorylation. Surprisingly, the reporter activity in the strain expressing ArcB_Hi_ was even higher than in the Δ*arcB* strain (Fig 5C), suggesting that under this aerobic growth condition, ArcB_Hi_ possess ArcA kinase activity, rather than the expected ArcA-P phosphatase activity. Thus, it appears that ArcB_Hi_ not only utilizes a distinct molecular mechanism for regulating its activity but also responds to different environmental growth conditions compared to ArcB_Eco_.

### ArcB_Hi_ responds to an aerobic metabolic signal

**Fig 6:**
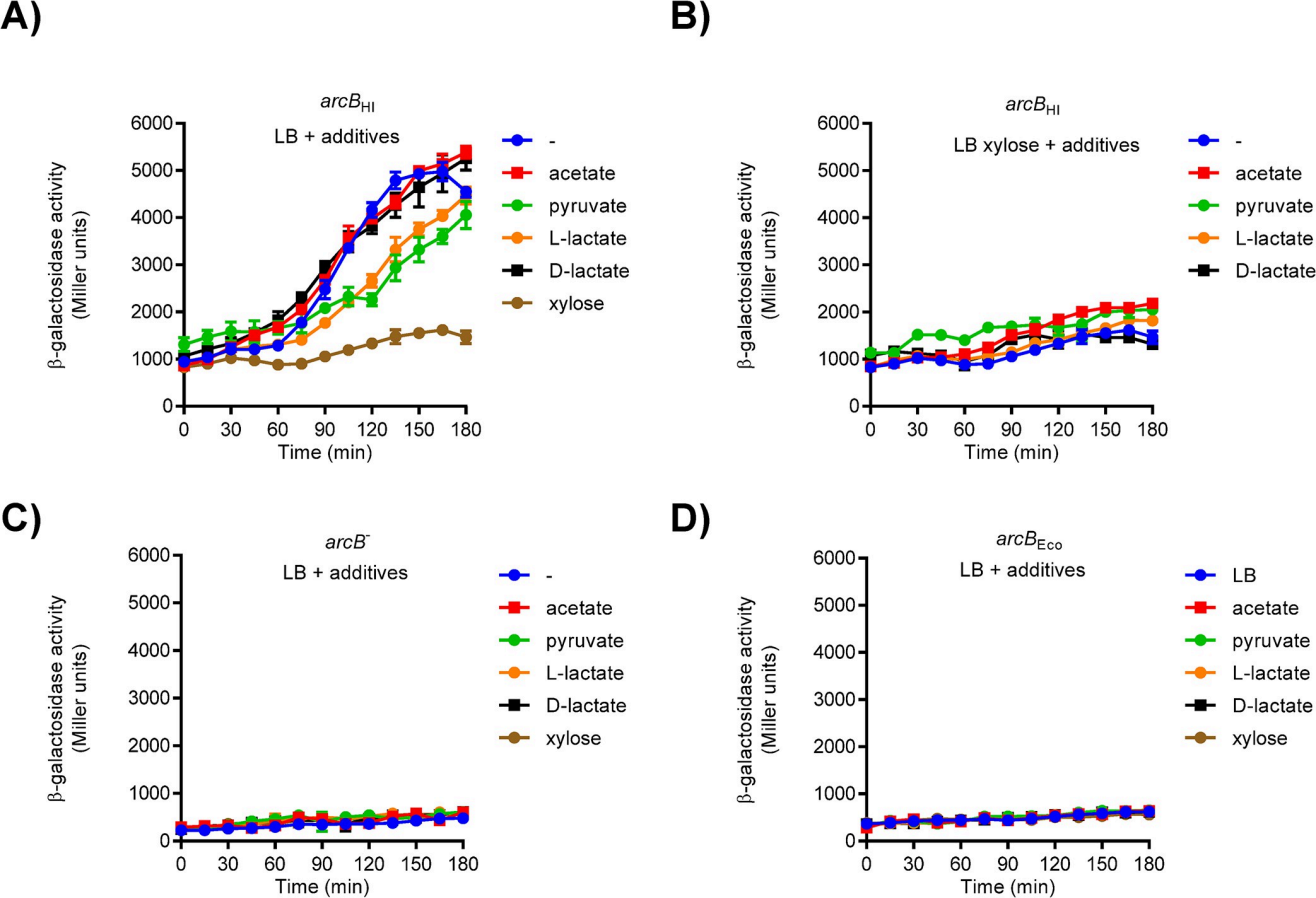
Effect of the addition of xylose and/or metabolic products on the aerobic activity of ArcB_Hi_. Cells of strains ECL5004 harboring plasmid pEXT22arcBHi (*arcB*_Hi_) (A and B), ECL5004 (Δ*arcB*) without plasmid (C), and ECL5003 (*arcB*_Eco_) (D), were grown aerobically in LB medium buffered with 0.1 M MOPS (pH 7.4), either unsupplemented or supplemented with 20 mM of acetate, pyruvate, L-lactate, D-lactate, or xylose. In panel B, 20 mM xylose was added alongside each of the other metabolic products. When the cultures reached an OD600 of 0.2, β-galactosidase activity was monitored for 180 minutes.

Our findings indicate that expressing ArcB_Hi_ in a Δ*arcB E*. *coli* strain restores ArcA phosphorylation and exhibits differential activity under aerobic and anaerobic conditions (Fig 1D). However, ArcB_Hi_ activation readily occurs even under aerobic conditions when pyruvate is the sole carbon and energy source (Fig 5C). Therefore, we argued that the ArcB_Hi_ activity might respond to a metabolic signal, rather than solely relying on oxygen availability. Indeed, L-lactate was previously proposed to be a potential activator of ArcBHi (Lichtenegger et al. 2014). Also, D-lactate, acetate and pyruvate were previously shown to act as physiological effectors able to amplify the autophosphorylation activity of ArcB_Eco_, and enhance the subsequent transphosphorylation of ArcA both *in vivo* and *in vitro* [16, 17]. We therefore tested whether the above-mentioned metabolic intermediates can promote ArcB_Hi_ activation under aerobic growth conditions. To do this, the ECL5004 (Δ*arcB* Δ*fnr cydA-lacZ*) strain harboring plasmid pEXT22arcBHi (*arcB*_*Hi*_) was grown aerobically in LB medium alone or supplemented with acetate, pyruvate, D-lactate, or L-lactate, and the time course of the reporter expression was followed (Fig 6A). Surprisingly, when cells expressing ArcB_Hi_ were grown aerobically in LB medium without additional carbon sources, reporter expression was activated at late exponential growth phase, reaching a steady state during the transition from exponential to stationary phase of growth. Addition of acetate, pyruvate, D-lactate, or L-lactate to the growth media did not significantly affect reporter expression (Fig 6A), suggesting that none of the tested metabolites can function as an effector/activator of ArcB_Hi_. On the other hand, addition of D-xylose prevented aerobic activation of *cydA-lacZ* expression (Fig 6A), as observed above (Figs 1D and 2) and in previous studies (Georgellis et al. 2001b). This observation explains, at least partially, the seemingly aerobic/anaerobic regulation of ArcB_Hi_ reported in both our previous [30] and current studies (Fig 1D), where D-xylose was included as a supplement in the growth medium. Interestingly, when cells expressing ArcB_Hi_ were grown aerobically in LB medium supplemented with D-xylose, addition of the aforementioned metabolites was not able to activate reporter expression (Fig 6B). Importantly, none of the above growth conditions induced aerobic reporter expression in either the Δ*arcB E*. *coli* strain (ECL5004) or the *arcB*_Eco_ strain (ECL5003) (Fig 6C and 6D), excluding the possibility of ArcB_Hi_-independent ArcA phosphorylation, and confirming the notion of aerobic activation of ArcB_Hi_. Thus, it appears that D-xylose impacts negatively ArcB_Hi_, and that none of the tested metabolites can act as the specific signal for ArcB_Hi_ activation. Although D-xylose had an unexpected inhibitory effect on ArcB_Hi_ activity, it should not be considered a silencing signal, since xylose did not suppress ArcB_Hi_ activity under anaerobic conditions. The exact mechanism by which xylose interferes with ArcB_Hi_ activation in aerobic environments is still being explored, as it could provide valuable insight for future studies on the activation mechanism of ArcB_Hi_. Taken together, our results suggest that the *H*. *influenzae* Arc system is activated during both aerobic and anaerobic growth conditions, responding to a yet unidentified metabolic signal, and that a distinct molecular mechanism governs the ArcB_Hi_ regulation.

## Discussion

The Arc TCS is a conserved regulatory pathway in γ-Proteobacteria, orchestrating cellular responses to environmental redox conditions. While the core architecture of Arc systems is generally conserved, our study on the *H*. *influenzae* ArcB reveals that the underlying regulatory mechanism vary significantly as compared to its *E*. *coli* counterpart.

Despite sharing substantial sequence similarity (36% identity and 54% similarity) with its *E*. *coli* homologue, we demonstrate that ArcB_Hi_ exhibits distinct regulatory features. Unlike *E*. *coli* ArcB, which is redox-sensitive due to the regulatory cysteine residues within its PAS domain [15], the PAS-less ArcB_Hi_ is not influenced by redox changes. Instead, we found that ArcB_Hi_ is activated during the transition from exponential to stationary growth phase under aerobic growth conditions, suggesting a metabolic signal-dependent activation mechanism.

Early studies reported that ArcB_Hi_ could functionally complement an *arcB* mutant strain of *E*. *coli*, mimicking its aerobic/anaerobic regulation [29, 30]. However, our findings indicate that such complementation only occurs under certain growth conditions. Indeed, aerobically ArcB_Hi_ activation is inhibited by the addition of D-xylose to the growth medium, suggesting that this ArcB ortholog may respond to metabolic cues. Our investigation revealed that common fermentation intermediates, such as acetate, pyruvate, or D-lactate, do not serve as direct activators of ArcB_Hi_. Additionally, L-lactate, previously proposed as a potential activator [40], was also ruled out in our study. The uncertainty of whether ArcB_Hi_ responds to an activating signal, an inhibitory signal, or both, as observed with ArcB_Eco_, complicates the prediction of a plausible stimulus for this sensor kinase. Nonetheless, it is tempting to speculate that factors such as the NADH/NAD ratio or the proton motive force (PMF), which fluctuate as cells transition from exponential to stationary growth phase and when switching between aerobic and anaerobic conditions [41–43], might influence ArcB_Hi_ activity. While the specific ArcB_Hi_ stimulus remains to be determined, it seems that the Arc systems of *H*. *influenzae* and *E*. *coli* have evolved to sense and respond to distinct environmental cues that are relevant to their respective lifestyles.

PAS-less ArcB homologs are prevalent in the Pasteurellaceae family, a group of specialized commensals or parasites of vertebrates with limited survival outside their host [30–32]. Members of this family, including prominent pathogens like *Pasteurella*, *Haemophilus*, *Mannheimia*, and *Actinobacillus* species, exhibit relatively small genomes, most likely a consequence of adaptive genome reduction associated with their parasitic lifestyle [44]. Due to this genomic streamlining, these bacteria typically encode only 4 to 7 two-component systems, with the Arc system being consistently conserved. Interestingly, a deletion event within the *arcB* coding sequence appears to have occurred in a Pasteurellaceae common ancestor, giving rise to PAS-less ArcB homologs, and it appears that these proteins evolved to acquire novel sensing capabilities while retaining the core signaling functions of the Arc system.

It is relevant to mention that in *Shewanella oneidensis*, a proteobacterium of the Altermonadales order, the Arc system shows a different evolutionary divergence. In this case, the *arcB* coding sequence is partially lost, retaining only the coding region of the histidine phosphotranfer domain (HptA), which appears to have co-evolved with ArcS, an ArcB unrelated hybrid histidine kinase, to mediate phosphorylation and activation of ArcA, regulating the expression of multiple genes involved in envelope stress response, oligopeptide transport, and translation [45–49].

In conclusion, our observations on the *H*. *influenzae* ArcB may illustrate a yet distinct regulatory mechanism, underscoring the diversity and adaptability of ArcB proteins across various bacterial species. The distinct regulatory features and physiological roles observed in these variants highlight the importance for further investigations to fully understand the functional nuances of these regulatory systems. Unraveling the precise nature of their activating signals, the underlying regulatory mechanisms, and their contributions to bacterial physiology and pathogenesis could provide valuable insights into bacterial adaptation and survival strategies.

## Supporting information

S1 Table NCBI reference sequence accessions of PAS-less ArcB orthologs used in multiple sequence alignment. (DOCX)
