# Supplementary material for: Diversification of signal identity and modus operandi of the Haemophilus influenzae PAS-less ArcB sensor kinase
Source: PLoS One. 2024 Dec 5;19(12):e0315238. doi: 10.1371/journal.pone.0315238 (PMC11620690; doi:10.1371/journal.pone.0315238)
Supplement: S1 Table — (DOCX) [file pone.0315238.s001.docx]

| Accession | Organism | Order | Family |
| --- | --- | --- | --- |
| WP_038440855.1 | *Haemophilus influenzae* | Pasteurellales | *Pasteurellaceae* |
| WP_111697046.1 | *Haemophilus haemolyticus* | Pasteurellales | *Pasteurellaceae* |
| WP_194812818.1 | *Rodentibacter haemolyticus* | Pasteurellales | *Pasteurellaceae* |
| WP_005696363.1 | *Haemophilus parainfluenzae* | Pasteurellales | *Pasteurellaceae* |
| WP_164028762.1 | *Rodentibacter caecimuris* | Pasteurellales | *Pasteurellaceae* |
| WP_100296657.1 | *Caviibacterium pharyngocola* | Pasteurellales | *Pasteurellaceae* |
| WP_095176874.1 | *Haemophilus pittmaniae* | Pasteurellales | *Pasteurellaceae* |
| WP_226690517.1 | *Pasteurella canis* | Pasteurellales | *Pasteurellaceae* |
| WP_078219058.1 | *Canicola haemoglobinophilus* | Pasteurellales | *Pasteurellaceae* |
| WP_077474236.1 | *Rodentibacter trehalosifermentans* | Pasteurellales | *Pasteurellaceae* |
| WP_094946599.1 | *Actinobacillus seminis* | Pasteurellales | *Pasteurellaceae* |
| WP_132023640.1 | *Bisgaardia hudsonensis* | Pasteurellales | *Pasteurellaceae* |
| WP_035685381.1 | *Avibacterium paragallinarum* | Pasteurellales | *Pasteurellaceae* |
| WP_109063893.1 | *Aggregatibacter kilianii* | Pasteurellales | *Pasteurellaceae* |
| WP_005764746.1 | *Pasteurella dagmatis* | Pasteurellales | *Pasteurellaceae* |
| WP_005761434.1 | *Pasteurella bettyae* | Pasteurellales | *Pasteurellaceae* |
| WP_011200898.1 | *Mannheimia succiniciproducens* | Pasteurellales | *Pasteurellaceae* |
| WP_006717417.1 | *Aggregatibacter segnis* | Pasteurellales | *Pasteurellaceae* |
| WP_116632432.1 | *Pasteurella langaaensis* | Pasteurellales | *Pasteurellaceae* |
| WP_126598614.1 | *Actinobacillus delphinicola* | Pasteurellales | *Pasteurellaceae* |
| WP_135709409.1 | *Actinobacillus porcinus* | Pasteurellales | *Pasteurellaceae* |
| WP_131977907.1 | *Cricetibacter osteomyelitidis* | Pasteurellales | *Pasteurellaceae* |
| WP_133542816.1 | *Mesocricetibacter intestinalis* | Pasteurellales | *Pasteurellaceae* |
| WP_132302890.1 | *Lonepinella koalarum* | Pasteurellales | *Pasteurellaceae* |
| WP_012072608.1 | *Actinobacillus succinogenes* | Pasteurellales | *Pasteurellaceae* |
| WP_064082813.1 | *Haemophilus ducreyi* | Pasteurellales | *Pasteurellaceae* |
| WP_006248378.1 | *Mannheimia haemolytica* | Pasteurellales | *Pasteurellaceae* |
| WP_078236731.1 | *Haemophilus paracuniculus* | Pasteurellales | *Pasteurellaceae* |
| WP_115314845.1 | *Phocoenobacter uteri* | Pasteurellales | *Pasteurellaceae* |
| WP_090921302.1 | *Pasteurella skyensis* | Pasteurellales | *Pasteurellaceae* |
| WP_005707381.1 | *Haemophilus parahaemolyticus* | Pasteurellales | *Pasteurellaceae* |
| WP_007527036.1 | *Haemophilus sputorum* | Pasteurellales | *Pasteurellaceae* |

**Table S1.** NCBI reference sequence accessions of PAS-less ArcB orthologs used in Multiple Sequence Alignment.
